# Examining the effectiveness of Second Step Early Learning versus free play on socio-emotional learning in kindergarten: a cluster randomized controlled trial protocol

**DOI:** 10.1186/s13063-025-09047-7

**Published:** 2025-08-26

**Authors:** Anders Dechsling, Christina Cipriano, Line Børtveit, Krister Westlye Fjermestad, Ann-Charlott Beatrice Amundsen Karlsen, Tore Marius Akerbæk, Charlotte Buer Tangen, Hugo Cogo-Moreira

**Affiliations:** 1 Department of Education, ICT and Learning, Østfold University College, Halden, Norway; 2 Child Study Center, Yale School of Medicine, New Haven, USA; 3 Department of Psychology, University of Oslo, Oslo, Norway; 4 Department of Computer Science and Communication, Østfold University College, Halden, Norway

**Keywords:** Cluster randomized controlled trial, Second step, Play, Socio-emotional learning, Empathy, Kindergarten, Academic achievement

## Abstract

**Background:**

This project evaluates the effectiveness of the Norwegian version of Second Step Early Learning (Nor-SSEL), a translated and culturally adapted Norwegian version of the American Second Step program, compared to free play in kindergartens, in a cluster-randomized controlled trial. Nor-SSEL is a socio-emotional learning (SEL) program that spans 28 weeks, emphasizing the development of learning skills, friendship skills, and emotional management. We hypothesize that Nor-SSEL is superior to free play in enhancing children’s social-emotional learning (SEL).

**Methods:**

We will allocate 990 children within 66 kindergartens, which will be randomized at the kindergarten level. The primary outcome is child psychosocial adjustment, assessed using the Strengths and Difficulties Questionnaire (SDQ), as reported by parents and teachers at baseline, immediately after the 28-week intervention in the kindergarten, and then 7 months after the transition to the new school (K1). The secondary outcome is empathy, assessed using the Empathy Questionnaire for Children (EmQue), which parents and teachers report at the same time points as the SDQ. Mediator analysis will be explored using the Joint Attention Scale, and as the moderator, we will analyze the parents’ socioeconomic status. A distal exploratory variable in follow-up assessment is academic achievement at the end of the first grade (math and reading skills). To reduce missing data, a web application will be developed to track children’s behaviors via teachers, parents, and children’s activities.

**Discussion:**

We believe that the Nor-SSEL impacts the joint attention score, which, in turn, affects the SDQ score. This study contributes to the scientific understanding of how the SEL curriculum intervention can impact SEL and future academic achievement in kindergarten-aged children. This could shape early education policies and practices in kindergartens, as well as their potential application to social, emotional, and academic skills.

**Trial registration:**

ClinicalTrials.gov NCT06975267. Registered on 16 May 2025. https://clinicaltrials.gov/study/NCT06975267.

## Administrative information

**Table:** Note: the numbers in curly brackets in this protocol refer to SPIRIT checklist item numbers. The order of the items has been modified to group similar items (see http://www.equator-network.org/reporting-guidelines/spirit-2013-statement-defining-standard-protocol-items-for-clinical-trials/).

Title {1}	The Effectiveness of the Norwegian version of Second Step Early Learning vs. Free Play on Socio-Emotional Learning in Kindergarten: A Cluster Randomized Controlled Trial Protocol
Trial registration {2a and 2b}.	ClinicalTrials.gov ID: NCT06975267
Protocol version {3}	May 2025, version 1
Funding {4}	The Research Council of Norway (RCN).
Author details {5a}	^1^Department of Education, ICT and Learning, Østfold University College, Halden, Norway ^2^Child Study Center, Yale School of Medicine, New Haven, USA ^3^Department of Psychology, University of Oslo, Oslo, Norway ^4^Department of Computer Science and Communication, Østfold University College, Halden, Norway

## Introduction

### Background and rationale {6a}

Students who participate in universal school-based social emotional learning (SEL) programs more often experience improved social emotional skills, prosocial behavior, academic achievements, and attitudes such as self-efficacy, self-esteem, emotions, and mindsets while experiencing reduced internalizing and externalizing problems [1]. SEL refers to the cognitive, affective, and behavioral skills and strategies that underscore human development and flourishing. A recent and extensive systematic review and meta-analysis of SEL programs [1] included 424 studies from 53 countries, reflecting 252 discrete universal school-based SEL interventions. The review included 575,361 students from kindergarten through 12th grade and two types of designs, randomized controlled trials (RCTs; 55.9%) and quasi-experimental studies (44.1%), available from 2008 through 2020. Most of the students were 5-year-old to 10-year-old children (56.8% of the 424 studies). Regarding the current protocol, only 24 out of the 424 studies were devoted to kindergarten (5.7%). The second step was the most commonly used intervention (*k* = 16).

Among other positive traits, academic achievement, better social relationships, communication skills, and increased confidence are associated with social and emotional competence [1–3]. In Norway, according to the Framework Plan for Kindergartens [4], inclusion and positive self-esteem are some of the main goals in kindergartens’ pedagogical fundament—democratic orientation, respect for each other, and knowledge regarding religious and cultural backgrounds. Since 2022, an expert group has examined how kindergartens and schools can contribute to ensuring that all children have equal opportunities in life, regardless of their background [5]. The expert group recently submitted their recommendations to the Minister of Education in Norway. The expert group’s goal was to propose measures that influence the well-being and health of children, as well as their academic and socio-emotional skills, while prioritizing socially redistributive measures. One of the main recommendations was systematic and targeted programs for social and emotional learning [5].

Our study is to be conducted in Norway. The Norwegian version of Second Step Early Learning (*Nor-SSEL)* is a translated and culturally adapted version of the American Second Step Early Learning (Cfc [6]). The Norwegian version is called *Småsteg—Steg for Steg for tidlig læring og ferdighetstrening i barnehagen* (Small steps – step by step for early learning and skill acquisition in the kindergarten). From now on, we will use the abbreviation *Nor-SSEL*. Among all the seven Norwegian RCTs evaluating the effects of an intervention on SEL [7–12], none of the studies involved kindergartens, regardless of the availability of *Nor-SSEL* in Norwegian, which has already been adopted in different kindergartens all over Norway, and only one evaluated the effect of the Norwegian *Second Step* [12]. Since that study involved a sample from upper elementary school (fifth to seventh grade) in 11 schools in Norway [12], we have no RCTs investigating the effectiveness of the *Nor-SSEL* in kindergartens in Norway. Despite this lack of demonstrated effects in the Norwegian context, unofficial data from the publisher of the *Nor-SSEL* showed that the *Nor-SSEL* had been delivered to more than 300 kindergartens.

In the Nordic countries, including Norway, there is a tradition for having a child-centered perspective where play is seen as essential for children’s holistic development (cognitive, emotional, social, and physical). Play is considered to provide a foundation for children’s development and educational outcomes. Some movements or traditions view assessments and structured interventions as unbeneficial to children, who should instead be engaged in ludic activities [13]. Several researchers and practitioners have argued that play in kindergarten is at risk of being minimized due to academic pressure [13–16]. Regarding learning, play has a key role. Play enhances the development of several important processes and is the basis for future learning in schools and employment. Children’s play affects motor development and social, language, and cognitive skills. This is why playing is a central point in the development of children [13]. The release of a recent report by an expert committee [5], which also recommended structured play as an important measure, has sparked a debate on the importance of free play versus structured play. In response to the report, prominent figures in the Norwegian context have argued that free play is “undoubtedly the best way to secure children’s social, cognitive, and emotional development” [17]. Considering these discussions, using play as the control condition will also generate more controlled data on the effects of play activities while safeguarding the ethical assumption regarding offering a non-harmful intervention as a control. The positive effects of a playful learning curriculum have recently been demonstrated in a Norwegian-based RCT [18]. A total of 1313 children in 96 kindergartens were randomly assigned to clusters in an intervention group (*n* = 49) consisting of a 9-month curriculum application and a control group (*n* = 47) continuing as usual. The analyses revealed a significant improvement in mathematical skills but not working memory or vocabulary [18].

### Objectives {7}

In the current trial, we plan to use a cluster randomized controlled design to compare the effectiveness of Nor-SSEL versus free play on SEL in kindergartens. The primary research question is: Is Nor-SSEL more effective than free play in enhancing children’s social-emotional learning (SEL) and empathy? Our hypothesis states that Nor-SSEL is superior to free play in enhancing children’s social-emotional learning (SEL) and empathy.

### Trial design {8}

This study is a cluster randomized controlled trial. The intervention group will receive Nor-SSEL for 28 weeks, and the control group will receive added playtime, with a 1-year follow-up (6 months after the transition from kindergarten to school).

## Methods: participants, interventions and outcomes

### Study setting {9}

The trial will be conducted in Norway, with kindergartens across the southeastern region prioritized in the cluster selection. The clusters represent each kindergarten in the two conditions. The intervention and control conditions will be implemented, and data will be collected from 66 kindergartens in Norway.

### Eligibility criteria {10}

To be eligible for this study, kindergartens cannot have implemented an active Nor-SSEL intervention in their kindergarten for at least the past 4 years from the start of this study and are currently “naïve” to Nor-SSEL implementation. The minimum number of 4- to 5-year-old participants we seek per kindergarten is 15. Children will participate in either the intervention or control condition as part of the pedagogical setting in the kindergarten, regardless of their involvement in the research study (i.e., data collection).

### Who will take informed consent? {26a}

This is a universal intervention study, and each kindergarten’s parents or guardians must consent to participate. The kindergarten employees will administer the consent form to parents who respond through the designated and secure web application. Author CBT is the main responsible for obtaining the informed consents, along with authors AD and HCM.

### Additional consent provisions for collection and use of participant data and biological specimens {26b}

In addition to the outcome measures, and based on parent consent, we will collect data on parents’ socioeconomic status as in Mekonnen et al. [19]. There are no biological specimens to be collected. The parents will be asked to provide additional consent for the storage and future use of their de-identified data in ancillary studies. They may withdraw their consent for future use without affecting their participation in the main study. All future use of data and samples will be subject to ethics committee evaluations.

#### Interventions

### Explanation for the choice of comparators {6b}

We have chosen an active control condition instead of a treatment-as-usual (TAU). TAU conditions in early childhood education settings will vary, and kindergartens may incorporate core elements of SEL interventions in the daily educational practices. Such variability can obscure the unique effects of the intervention and thus have implications for the interpretation of effectiveness [20]. This has been demonstrated in research in other clinical fields, where overlap between TAU and the intervention can diminish apparent treatment effects [21, 22]. An active control allows for a comparison condition that is better described and more consistently focuses on the effects of the intervention itself. Given that the majority of SEL trials to date have relied on TAU or waitlist controls, in Cipriano et al. [1], 72.9% (381) of the studies used TAU or waitlist controls; the use of an active control group in this study contributes to advancing methodological standards in the field.

After carefully considering an appropriate control arm, we decided on a designated play time for the same daily duration as the Nor-SSEL program. Kindergartens in both conditions will keep the same amount of playtime as prior to the study, but for the time the Nor-SSEL kindergartens spend on Nor-SSEL, the control kindergarten spends the same time on free play. This means that this particular play time is added to the regular play time in the kindergartens. The active control kindergartens will therefore have additional designated playtime (equivalent of time spent on Nor-SSEL) for free play for 28 weeks, creating a methodological and meaningful comparator to Nor-SSEL. The playtime will have the same daily duration as the intervention program and thus replace time spent on e.g., educational or other activities.

We have decided that “free play” must be defined, as the same activity can be perceived as play one day and not the next. This means the child’s behavior is the same, but the context and experience differ. Additionally, it is subjective to determine what is experienced as play and what is not. Therefore, we do not have special requirements for what characterizes “play” and “proper play” but instead describe conditions that must be present to be defined as a situation facilitated for play. A checklist defining the play condition was developed and evaluated by five experts on children’s play in the kindergarten context.

The checklist for play conditions is defined as follows: Time is set aside in the daily kindergarten routine where (1) it is clearly announced or signaled to the children that it is now “free play”; (2) there is an absence of requirements for sedentary sitting (unless it is part of the children’s own initiated play); (3) it is not a situation where children are required to follow instructions (unless it is part of the children’s initiated play); (4) there is an absence of a pronounced and specific learning outcome or goal with the activities available for the children; (5) the children can start and finish activities themselves and choose what they want to participate in; and (6) adults can join in the game/initiate, but on the children’s terms, and the children can choose to leave this game whenever they want. Children requiring assistance to engage in play will receive structured support [16, 23].

One teacher and a manager from each kindergarten will attend an orientation session with experts on children’s play to discuss and provide a mutual understanding of the premises of the play condition. This session is to provide an equivalent amount of attentional focus as for the ones in the intervention condition. The teachers will have simulation training and also a specific workshop on assisting children who struggle with engaging play. The entire staff of each kindergarten, teachers and assistants involved with the children in each kindergarten, will be introduced to the play checklist via the web application and receive training from the teachers who have attended the session. In addition, our team will purchase toys or equipment for the kindergartens in the control condition, with a value of up to 11,000 NOK per kindergarten, intended to stimulate play. Such purchases are made via designated people in the research team to ensure compliance with the intended use.

### Intervention description {11a}

The intervention *Nor-SSEL* [24] emphasizes learning skills, friendship skills, and managing feelings. The intervention lasts 28 weeks, is divided into five themes, and consists of daily lessons. The themes are (1) Skills that promote learning, (2) Empathy, (3) Managing emotions, (4) Friendship skills and problem solving, and (5) Transitioning to school. The intervention consists of daily activities. The guidebook is a central component of the program, providing kindergarten teachers with practical tools and activities to implement the program in everyday settings. The guidebook includes 28 weekly themes. A new theme with related activities and discussion topics is introduced each week. Brain-building games and activities are designed to strengthen children’s cognitive skills and self-regulation. Furthermore, the guidebook includes songs and music intended to reinforce the learning of social skills, as well as information sheets and activities that engage parents in their children’s social-emotional development.

One teacher and a manager from each kindergarten will attend a training session with experts responsible for the program’s translation and adaptation from the start, and with more than 20 years of experience with the Nor-SSEL. During training, teachers take turns leading various sections in groups and have contact with different components of the program (e.g., executive function skills, wait time, think/turn/tell). The entire staff of each kindergarten, teachers and assistants involved with the children in each kindergarten, will be introduced to the Nor-SSEL via the web application and receive training from the teachers who have attended the 2-day training session, and use the online training resource simultaneously during regular working hours in planning sessions.

### Criteria for discontinuing or modifying allocated interventions {11b}

Parents may withdraw their consent at any time without affecting their participation in the main study; however, the intervention and control conditions are part of the kindergarten’s core educational activities. If needed, kindergartens and parents need to seek alternative arrangements for the child during the intervention/control activity. Kindergarten staff will report instances where the study is suspected to worsen the child’s functioning and well-being, in which case the project’s designated clinical specialist will be contacted.

### Strategies to improve adherence to interventions {11c}

We use teacher logs to track the loading and implementation of the Nor-SSEL and the “free playgroup.” We will develop a specific formulary for monitoring the activities developed in the control condition. A web application is developed for collecting data per the outcome measures based on children’s behaviors via teachers and parents, replacing the manual reporting process and collecting data for the trial. The web application helps streamline reporting and data collection for teachers, parents, and researchers. The app facilitates connections between various data points and archives information about children over time.

Furthermore, the app adopts a module-based structure mirroring the Nor-SSEL program’s curriculum. Weekly questionnaires linked to specific modules align with themes. The app tracks the completion status, generating statistics for future reference. Module access is dynamically controlled, opening and locking based on completion to ensure adherence to the Nor-SSEL curriculum order. This allows robust analyses of the adherence effect via compiler-average causal analysis. For the control condition, given that there is no pre-structured program, teachers will report daily amounts of free play.

The key web application development elements are (1) data collection, (2) role-based access, (3) module-based curriculum order, (4) push notifications, (5) educational materials, and (6) user experience and data collection efficiency. The development process follows a User-Centered Design approach [25] involving teachers, parents, and researchers in decision-making during the pilot phase of the RCT. This collaborative methodology spans several stages: (1) preliminary examination (e.g., user understanding, reviews, data protection measures, expert interviews); (2) conducting expert interviews with software developers and stakeholders; (3) heuristic analysis for usability (e.g., analyzing other modular-based designs for robust guidelines); (4) user understanding (e.g., generating and utilizing personas, scenarios, etc., to understand user types); (5) prototype development (low fidelity through co-creation workshops, usability testing, user feedback, and high fidelity type for technological aspects and functions); (6) usability testing (based on Krug [26]); (7) security measures (e.g., the General Data Protection Regulation [GDPR]) use authentication and authorization protocols, data encryption and backups, legal agreements, and user manuals and agreements to address potential human errors.

### Relevant concomitant care permitted or prohibited during the trial {11d}

To ensure the internal validity of the study, participating kindergartens must not concurrently implement other structured programs targeting similar domains of children’s social-emotional development. Such programs share overlapping objectives with Nor-SSEL, particularly in promoting empathy, emotion regulation, and prosocial behavior. There is no need for permitting or prohibiting any other concomitant care or interventions.

### Provisions for post-trial care {30}

Our team has a clinical specialist in child and adolescent psychiatry, specialized in early childhood mental health, who will provide support during the study, dealing with situations where atypical scores not previously reported by the parents could indicate that children are at risk for mental health problems. The specialist will be available throughout the study, i.e., for at least 1 year following intervention completion. The risk of participants experiencing adverse events during this trial because of the interventions is very low. However, we will employ safeguarding procedures to mitigate the risks of adverse effects by the specialist in child and adolescent psychiatry. The responses in the SDQ will be screened for atypical scores indicating risk for mental health problems. Where any participant discloses information that shows them or others to be at risk of significant harm, the research team will make direct referrals to relevant service providers. The referral process will be triggered through survey responses that indicate potentially harmful experiences and/or support needs (e.g., high scores on the SDQ scale). Direct referrals to in-person social services will also be available if parents request them to do so.

### Outcomes {12}

The primary outcome is child psychosocial adjustment measured using the *Strength and Difficulties Questionnaire* (SDQ; [27]), which will be answered by at least one of the parents and kindergarten teachers at baseline (T1), immediately after the 28 weeks of intervention (T2), and then 6 months after (T3) the transition to school (K1). The SDQ comprises 25 items and assesses five dimensions: emotional symptoms, conduct problems, hyperactivity/inattention, peer relationship problems, and prosocial behavior, which are the most common outcomes of SEL [1]. We will use the general continuous score for the analysis.

The secondary outcome is empathy, which at least one of the parents and teachers measures via the Empathy Questionnaire for Children (EmQue) toddler version. The questionnaire comprises 19 items assessing emotional contagion, attention to others’ feelings, and prosocial actions [28]. The tests are composed of two domains: cognitive and affective empathy. We will use EmQue’s general raw continuous score.

The exploratory distal outcome will measure the academic achievement score (last month of the first grade) in reading and math, represented by the standardized assessment tests (in Norwegian called *Kartleggingsprøver*) developed by the Norwegian Directorate for Education and Training [29]. The results of the tests are continuous scores for both reading and mathematics.

#### Moderators and mediators

The moderator will be the parents’ socioeconomic status (SES). Parents will self-report the same items as in [19], namely the education levels (years of schooling) and the household incomes. SES plays an important role in shaping children’s socio-emotional trajectories, and children from lower SES backgrounds are found to face increased difficulties throughout school compared to their peers [30]. Given the disparities between children from different socioeconomic statuses (SES), it may moderate the effectiveness of socio-emotional learning (SEL) interventions. By including SES as a moderator in this randomized controlled trial, we will be able to examine whether the program has an impact across social strata, ensuring that the findings are not only generalizable but also sensitive to issues related to SES.

The mediator is a continuous joint attention score, considered a predictor of subsequent functioning in the social, communicative, and cognitive domains [31], where the kindergarten teachers will be trained [32]. The Joint Attention Scale consists of six naturalistic examiner-initiated prompts designed to elicit a response to joint attention. The available protocol for joint attention will be translated and adapted for use in Norway. As a mediator, its role in the modeling will allow us to explore how (mechanism hypothesis) the effects of the intervention might impact the outcomes.

### Participant timeline {13}

**Table:** 

	**STUDY PERIOD**					
	**Enrolment**	**Allocation**	**Post-allocation**			
**TIMEPOINT**	*** − t***_***1***_	**0**	***t***_***1***_	***t***_***2***_	***t***_***3***_	***t***_***4***_
**ENROLMENT:**						
**Eligibility screen**	X					
**Informed consent**	X					
**Teacher training**		X				
**Allocation**		X				
**INTERVENTIONS:**						
**Intervention:** **Second Step Early Learning (NOR ed.)**						
**Control condition: Free play**						
**ASSESSMENTS:**						
**SDQ** **(primary outcome)**			X		X	X
**EmQue** **(secondary outcome**			X		X	X
**Joint Attention Scale (mediator)**				X		
**Caregiver SES (moderator)**			X			
**Std. 1st grade screening test**						X

#### Sample size {14}

Our sample will consist of 990 children aged 4 to 5 years old, distributed in groups of > 15 per kindergarten. Our sample size calculation considered the following input parameters: (1) two groups in a cluster design with an allocation ratio of 1:1; (2) a minimal detectable effect size difference of *d* = 0.27, which is the upper limit confidence interval for the effect size reported on SEL skills derived from 114 studies [1], with a mean of 0.219 (95% CI = 0.171–0.267). In this meta-analysis, the most common measure used to track SEL was the *Strength and Difficulties Questionnaire* [27], which is our primary outcome measure and rated by teachers and parents. Such a difference of 0.27 is described as a small effect size, according to the traditional benchmarks proposed by Cohen [33]. When considering only the effect sizes among the youngest (called by Cipriano et al. [1]) as elementary schools (Kindergarten [5 years old] to 5th grade [10 years old]) from exclusively randomized controlled trials and using SEL skills as outcomes, the average adjusted effect size at post-assessment was 0.241 (derived from 96 effect sizes). Therefore, our minimum expected effect size is closer to the state-of-the-art. However, the effect sizes depend on the population, outcome, and nature of the implemented intervention. Researchers have recently used data from systematic reviews and meta-analyses to recalculate the cutoffs for the difference [34–36]. (3) Intraclass coefficient correlation-derived SDQ domains ranged from 0.015 to 0.064 [37]; (4) Power was 0.8, and the adopted significance level was 0.05.

A parallel, two-group cluster-randomized design will be used to test whether the Nor-SSEL (active intervention) mean is different from the Group “play” (active control) mean. The comparison will be made via a two-sided *t*-test with degrees of freedom based on the total number of subjects [38, 39], with a Type I error rate (*α*) of 0.05. The common subject-to-subject standard deviation for both groups is assumed to be 1, the intracluster correlation coefficient is considered to be 0.065, and the coefficient of variation of cluster sizes is assumed to be 0.65. For a power of 80%, with 30 kindergartens of 15 subjects per kindergarten in Group *Nor-SSEL* (totaling 450 children) and 30 kindergartens of 15 children per kindergarten in Group *Play* (totaling 450 subjects), the detectable mean difference (μ1 − μ2) is 0.27. Therefore, 900 children in 60 kindergartens are needed. To compensate for the potential decrease in power due to attrition, we will recruit 990 children from 66 kindergartens. The detectable difference was computed using PASS version 23.0.1.

### Recruitment {15}

We have a designated team that actively recruits kindergartens through various means, including letters, emails, advertising, student networks, and other methods, until we reach the calculated number of kindergartens. This approach involves contacting municipalities and inviting as many kindergarten leaders as possible to a gathering workshop scheduled for March 2026. During this workshop, we will introduce the project’s aims and team. Ultimately, those leaders interested in participating in the study will sign up for an intention list. If more than 66 leaders show interest, a random selection will be used to select only 66, and then a randomization for each arm of the study will be conducted in front of every individual using a true random generator. If fewer than 66 leaders sign up, a new event will be scheduled in a different region until the number of 66 is achieved.

#### Assignment of interventions: allocation

### Sequence generation {16a}

We will use simple randomization at the kindergarten level. A random list of 66 elements will be created externally via Efron’s biased coin [40] implemented in PASS, where half of the numbers will be Nor-SSEL and the other half will have additional play time.

### Concealment mechanism {16b}

Our team of recruiters will lead the workshops and will be blinded to a random list of 66 elements created by someone external to the Norwegian team before the workshops. This list will be kept in a sealed envelope stored in a safe in the USA at Yale University. After the workshop with the kindergarten leader, the kindergarten names will be organized into a list. Kindergarten leaders will return to their kindergartens to discuss the project with parents. After parents’ approval and the baseline assessment, with a minimum of 15 children, is completed by the parents and teachers, the kindergarten and research team will receive a notification about the assignment status based on the previously randomized sequence order and the completion of the baseline assessment via our online system. The system is automated to identify when the information of the 15 children is available, and only after that will the system assign the arm status based on a randomly generated list. Recruiters, hence, cannot predict the next allocation. Simple randomization and blinding of the recruiters are intended to eliminate selection bias [41].

### Implementation {16c}

After our workshops, we will send the names of the kindergartens to the software developer, ACBCK, and TMA to register the potential kindergartens included in the system. Then, a notification will be sent to the kindergarten leaders, notifying them that they are invited and requesting their consent from parents. As soon as parents’ consent to their children’s participation, the baseline assessment of outcomes is implemented in the system, among parents and teachers. These steps and procedures ensure that the baseline assessment is fully completed before randomization, thereby avoiding the implementation in the kindergarten with incomplete baseline data. Once the data from the 15 children per kindergarten is completed, the system will allocate the random sequence previously generated by CC. Recruiters, hence, cannot predict the next allocation. The system will display, in real time, the project team and other kindergartens participating, the number of kindergartens that have already received an assignment (without disclosing the Nor-SSEL or additional playtime status) and the number of available spots for participation in the study. This measure aims to demonstrate the transparency of the study recruitment process, given that we have limited spots available. Additionally, this procedure will prevent delays in recruitment, motivate the kindergartens to submit their data as soon as possible, and allow them to be included promptly.

## Assignment of interventions: blinding

### Who will be blinded {17a}

Researchers will be blinded to the link between kindergarten and the assigned randomization until the baseline is assessed. After that, the research team will know the kindergarten’s random status and allocate them to receive the training in the Second Step. Blinding informants in this context is not possible. Participants are not masked to the intervention status; teachers and parents are the primary informants of the outcomes.

### Procedure for unblinding if needed {17b}

N/A: Since blinding only occurs at the point of data analysis, there is no need for unblinding.

#### Data collection and management

### Plans for assessment and collection of outcomes {18a}

Only quantitative data, based on scales and questionnaires, will be collected throughout the one-and-a-half-year follow-up period. All outcomes of intervention effectiveness will be measured at baseline, immediately after the 28 weeks of the intervention, and 7 months after transitioning to the first grade (approximately 1 year after the intervention’s completion). Math and reading grades will be collected at the end of the first grade.

### Plans to promote participant retention and complete follow-up {18b}

Parents and teachers will receive notifications about the pending information throughout the evaluation period, which will last for one and a half years. The research group will maintain direct contact with the kindergartens, discuss our progress in organizing online workshops and in-person seminars, and report on our progress between assessment waves to engage parents.

### Data management {19}

The study activities ensure that research data are stored, backed up, managed, and curated securely, in line with the GDPR, national data protection legislation, and local institution data protection policies. All the data will be collected through the web application. Therefore, there is no transportation of paper data in sealed envelopes, locked boxes, or in locked cabinets. Questionnaires and consent will be administered via password-protected devices by teachers and parents at the field site (kindergarten and at home), and data will be automatically uploaded into a secure server through the officially approved web-based survey tool *Nettskjema*, which ensures proper data protection services [42]. The data will be deidentified before analysis to ensure the confidentiality of the participants, with identifiers kept separate from the research data and linked through unique codes.

Personal information—such as name, email address, and role in the project (e.g., parent, teacher, or kindergarten student)—will be stored separately (in Nettskjema) from the questionnaire data. Children’s and parents’ full names will be used as the basis for generating a unique identifier for each child, which consists of eight characters. This code will be integrated by matching using the Levenshtein algorithm, which can identify similarities between a set of characteristics to match children’s assessments over time without disclosing sensitive information [43]. This code enabled the researchers to match individual questionnaires from different evaluation time points and raters (parents and teachers’ assessment) while maintaining the anonymity and confidentiality of the participants, which is essential for a study involving such sensitive data on children’s behavior [44]. Once the data collection phase of the study is complete, the form containing personal information will be deleted to ensure that questionnaire responses cannot be traced back to individual participants.

Nettskjema is the primary data storage platform and will be integrated into the web application via the Nettskjema API. All data collected through the web application will be submitted directly to Nettskjema as form entries and stored accordingly. Electronic records from data collection will be exported from the respective platforms to secure servers at Nettskjema. Non-electronic data, such as letters of intention to participate from the kindergartens, will be stored in locked fire-safe 6-digit filing cabinets at Østfold University College.

### Confidentiality {27}

We will use a unique participant number for the research data instead of the names of the children, parents, and teachers. Personal identifiers will be collected only for informed consent, operational needs, and logistics (e.g., participant tracing by intervention staff or during follow-up visits). Personally identifiable information (e.g., names and phone numbers) will not be shared with other organizations outside of the research team. It will be destroyed after the study unless it is required for follow-up and/or future studies, and parents have consented to this. Confidentiality will be maintained by delinking all personal identification from the final datasets used for analysis. All anonymized data will be retained for 5 years, per the following ethical standards, and all non-anonymized data will be disposed of upon completion of the study. Since only de-identified quantitative web application engagement data are provided to the research team, we foresee minimal risks to participants.

### Plans for collection, laboratory evaluation, and storage of biological specimens for genetic or molecular analysis in this trial/future use {33}

N/A: No biological specimens will be collected in this study.

#### Statistical methods

### Statistical methods for primary and secondary outcomes {20a}

Due to the longitudinal (measured by three repeated raw continuous scores) and multilevel design (children nested in schools), two multilevel mixed-effects models will assess the intervention’s effectiveness on primary and secondary outcomes. We will use the maximum-likelihood estimator and, therefore, invoke the full-information maximum likelihood method to address missing data, assuming a missing mechanism of missing at random. The residuals will be a priori specified with a first-order autoregressive structure to account for within-subject correlation across time points (three repeated time points).

For all outcomes, an unstructured covariance matrix will be used to estimate the covariance between the random intercept (baseline assessment of the outcome) and random slope for time (i.e., potential speed of the improvement on the outcomes) at the children-level, allowing for maximal flexibility in modeling their relationship without imposing symmetry or independence.

Depending on the distribution of the outcomes, non-linear models can be implemented; however, we do not have a specific a priori model for this approach, as it is necessary to evaluate the distributional features of the observed data.

### Interim analyses {21b}

N/A: No interim analyses are planned for this study.

### Methods for additional analyses (e.g., subgroup analyses) {20b}

We plan to test two mediation hypotheses: (a) random assignment will impact the joint attention score (continuous score), which in turn will impact the SDQ general score; (b) long-term distal and serial effects where random assignment will impact the joint attention score (continuous score), which in turn will impact the SDQ general score, which, lately, will impact the math and reading scores at the end of the first grade. Mediation analysis will be conducted under the counterfactual framework, where the models offer flexibility in estimation due to the exposure–mediator interaction [45], allowing the effects to be decomposed as described by VanderWeele [46].

We also plan to study the effectiveness of the intervention on the SDQ and EmQue subscales via a latent approach, where the CT-C(M-1) model [47] will be employed to disentangle methods (at least one of the parents and teachers) and trait effects (five domains of the SDQ and three domains of the EmQue) in the post-intervention assessment. We plan to run two different types of models: one considering the 50 polytomous items in total for the SDQ (i.e., 25 from teachers + 25 from parents for the SDQ) and 38 items for EmQue (19 teacher + 19 parent items for EmQue), given our robust sample size to use diagonally weighted least squares (WLSMV) as an estimator (the default estimator for ordinal observed variables). The other modeling approach is to create five parcels, one per SDQ domain, in the case of EmQue, or three parcels, as suggested by Eid et al. [47], which generates a more parsimonious model [7]. Therefore, given that our “items” are now five (SDQ) or three (EmQue) approximations of continuous outcomes, for the CT-C(M-1) model, we will use maximum likelihood with a robust standard error estimator. Within each domain, the balancing approach was used to create parcels (see “single factor analysis” parceling, Landis et al. [48]), where the item with the highest item-scale correlation is paired with the item that has the lowest item-scale correlation. The advantages and disadvantages of parceling have been discussed in several contributions [49, 50].

Finally, we examine the interplay between the SDQ and EmQue scores using the latent change score approach [51] to understand whether empathy influences SEL skills (or vice versa). The adopted statistical significance level will be 0.05, and all analyses will be conducted using Mplus version 8.11 [52].

### Methods in analysis to handle protocol non-adherence and any statistical methods to handle missing data {20c}

Depending on the assumed missing data mechanism, two robust models will be considered to address missing data, specifically missing data at random (multiple imputation; see Taljaard et al. [53]) and missing data not at random (selection model; [54]).

To address a lack of adherence (i.e., kindergartens are randomized to receive the *Nor-SSEL* but still need to implement it integrally over the 28 weeks), we will use an approach called the Complier-average causal effect [55, 56], which gives an unbiased estimation of the effect of the intervention when nonadherence under different assumptions is considered (see Little & Yau [57]), especially under a multilevel design [50], as in the current study.

### Plans to give access to the full protocol, participant-level data, and statistical code {31c}

All components of this research, including but not limited to the research protocol, fully anonymized datasets, code, and data analyses, will be made available on the Open Science Framework. Project outcomes will be shared through peer-reviewed publications and actively disseminated to government officials, policymakers, NGOs, and communities within Norway and globally.

#### Oversight and monitoring

### Composition of the coordinating centre and trial steering committee {5d}

The Trial Scientific Steering Committee (TSC) will include all the authors listed in this protocol paper. All the authors will be responsible for the scientific conduct of the trial under the leadership of Author HCM. Additionally, a Trial Project Management Team will oversee the trial’s day-to-day operational requirements. Additionally, chaired by HCM, this team will include the first, fifth, sixth, and seventh authors, as well as at least one additional senior adviser from the Section for Research Administration at Østfold University College.

### Composition of the data monitoring committee, its role and reporting structure {21a}

The project management acts as the data monitoring committee, which is led by the fifth author. Deidentified data will be made available upon request. There are no competing interests or sponsor conflicts.

### Adverse event reporting and harms {22}

This RCT is considered low-risk, non-invasive, and non-clinical. It is implemented by kindergarten teachers in a class environment where activities are planned and discussed with the research team as per the protocol. Teachers can cease implementation at any time if they consider the program to be ineffective or if it creates undue harm. Furthermore, no adverse harms were reported in the cluster of RCTs involving SEL skills in the systematic review and meta-analysis by Cipriano et al. [1].

### Frequency and plans for auditing trial conduct {23}

Our project has no independent auditing process. A project management team and data management team, composed of a multidisciplinary team of experts (biostatisticians, educators, computer science experts, and kindergarten teachers), meet biweekly and hold joint monthly meetings to monitor trial conduct and data quality. The TSC provides oversight through its annual meetings.

### Plans for communicating important protocol amendments to relevant parties (e.g., trial participants, ethical committees) {25}

Substantial protocol amendments will be communicated to the research ethics committee of the Østfold University College, as well as the TSC and relevant online trial registry. Should such amendments concern study participants, they will be communicated through the local implementing partners, and additional consent will be sought where necessary. Non-substantial amendments will be kept on record.

### Dissemination plans {31a}

The results will be disseminated through academic publications to ensure peer review of the findings and their interpretation; we anticipate three main publications: (1) the effectiveness manuscript focused on the effects of the intervention on the outcomes and the distal exploratory measure, (2) the mediation focused manuscript targeting a closer and longer effect of intervention (based on serial mediation modeling), and (3) a cross-lagged panel model effect between empathy and SEL skills. The findings will be widely disseminated within Norway to education and health policy audiences, participating schools, and relevant professional associations. International dissemination will be achieved primarily through academic conferences and publications.

## Discussion

We believe that the Nor-SSEL will impact the SDQ and EmQue scores and the joint attention score. This study contributes to the scientific understanding of how such interventions can impact SEL and future academic achievement in kindergarten-aged children and provides insights into mechanisms through which interventions such as Nor-SSEL in early childhood might aid in the development of SEL. This could shape early education policies and practices and even their potential transposition to academic skills in the first grade. This study involves several logistical and coordination challenges, particularly related to participant recruitment and the risk of attrition during the transition from kindergarten to school. Strategies have been designed to address potential challenges in recruiting kindergartens and children through active recruitment until we reach sufficient participation.

Delivering the Nor-SSEL consistently across study sites requires thorough personnel training and close monitoring. Standard operating procedures following the guidebook and the web application are in place to support fidelity to the intervention protocol. Additionally, thorough training and follow-up will be provided to the active control condition to support the integrity of the condition. Site coordination is managed through biweekly project management meetings and monthly cross-functional team meetings. This structure is intended to facilitate communication and early identification of potential issues.

Missing data and data management are issues we have addressed by robust data quality assurance procedures using a web application with timely prompts to ensure data completeness and accuracy. Using the web application and Nettskjema, we seek to streamline data collection while minimizing the burden on staff and parents.

Specific attention has been given to ensuring ethical compliance and participant understanding. Input from community representatives, professionals, ethics advisors, and researchers has been integrated into the study. The control condition is considered meaningful, considering the ongoing discussion in the Nordic setting on using standardized pedagogical programs as part of the core educational activities.

### Trial status

Protocol version number: 1. June 4th 2025. Not yet recruiting. Recruitment begins August 2025 and will be completed by June 2026.

## Data Availability

Data sharing is not applicable to this article, as no datasets were generated or analyzed. Some datasets generated by the study will be made available via an open-access repository once fully anonymized and after the main study findings have been published.

## Abbreviations

No-SSEL: Norwegian version of Second Step Early Learning
SEL: Socio-emotional learning
RCT: Randomized controlled trial
SDQ: Strength and Difficulties Questionnaire
EmQue: Empathy Questionnaire for Children
RCN: Research Council of Norway
SES: Socioeconomic status
GDPR: General Data Protection Regulation
WLSMV: Diagonally weighted least squares
TSC: Trial Scientific Steering Committee
PRE-FER: Institutional ethical advisory commission at Østfold University College
REC: Regional Ethics Committee
